# Proved by Its Exceptions

**Published:** 1868-06

**Authors:** 


### PROVED BY ITS EXCEPTIONS.

"If an architect were to elaborate plans and drawings of a superstructure with the foundations contracted to dimensions much less than the upper stories, the universal verdict would be either mental imbecility or insanity. It is equally as impossible to rear a mental or a moral structure upon a feeble or sickly organization. Therefore a robust physique is indispensable to the origination of either mental or moral power."

That without a "robust physique" a man can have neither mind nor morals is a little rough on the invalids; but, as "the exception proves the rule," we must admit that this one has so many facts arrayed against it, that it stands beyond all dispute.

So we must conclude that David didn't kill Goliath and those other wild beasts, nor write the Psalms; for such an invalid as the 38th and 119th Psalms describe, hadn't the mental and moral power necessary for such performances. Paul's epistles inculcate pretty sound morals, and it is doubtful if Gamaliel found him a dull pupil; yet he was "in bodily presence weak." Indeed, there are several exceptions: Lindley Murray's school books were hard to displace, yet they were written in bed. Dr. Johnson, Cowper, Pope, Hood, Calvin, and Kirke White, all originated both mental and moral power, yet all were invalids. "Stonewall" Jackson didn't show much lack of mental power; and Alex. Stephens has some reputation for learning and statesmanship, though not strong enough to draw his last breath. And, to come nearer home, the member of our own profession who, for a dozen years, has done, and who is still doing more than any other to advance the cause of Dental science and progress, is a sickly little cripple who never took time to imagine what a "robust physique" would feel like.

It may be that the statement in our text is a little too strong; but a strong man made it. A few years ago, a theological student, on trial before the Board, began a discourse as follows: "There is always something sublime in the dying words of men!" When criticism was called for, a gray haired father thought the first sentence a little too strong. "It might do to say there is usually, or often; but to say there is *always*, might be objected to, as only last week Bill Poole died with the words, 'I'M A GONER.'"

This is written, not for science, but solely to cheer the invalids. With suicidal mania prevailing as an epidemic, statements like our text are dangerous. Persuade a clever invalid that the best he can hope for is to be both a fool and a rascal, and there is no telling what he might do.

The speaker needs yet to study the first principles of the physiology of the nervous system. W.

